# Feedback on Trait or Action Impacts on Caudate and Paracingulum Activity

**DOI:** 10.1371/journal.pone.0129714

**Published:** 2015-06-23

**Authors:** Alva Appelgren, Sara L Bengtsson

**Affiliations:** Department of Clinical Neuroscience, Karolinska Institutet, Stockholm, Sweden; Zhejiang Key Laborotory for Research in Assesment of Cognitive Impairments, CHINA

## Abstract

There is a general conception that positive associations to one’s trait, e.g. ‘I’m clever’, are beneficial for cognitive performance. Scientific evidence shows that this is a simplification. In this functional magnetic resonance imaging (fMRI) study we used written trial-based trait feedback ‘you are clever’, or task feedback ‘your choice was correct’, on each correct response of a rule-switching task, to investigate how the character of positive self-associations influences performance outcome. Twenty participants took part in this crossover design study. We found that trait feedback was less beneficial for motivation and performance improvement, and resulting in enhanced neural activation on more difficult bivalent rule trials. This indicates that the task was treated as more complex in this condition. For example, ‘you are clever’ feedback led to enhanced activation in anterior caudate nucleus, an area known to process uncertainty. We further observed that activation in anterior paracingulate cortex was sensitive to whether self-reflection was imposed by external feedback or generated from internal processes, where the latter activation correlated positively with performance when following after task feedback. Our results illustrate how feedback can evoke self-reflections that either help or hinder motivation and performance, most likely by impacting on processes of uncertainty. The results support social psychological models stipulating that trait focus take resources away from task focus.

## Introduction

Self-reflection is an inherent trait in humans and is there to enable predictions about one’s behaviour so that one can regulate attention and make informed choices [1]. In a review by Passingham et al., [2], several examples from animal and human brain imaging studies were presented which showed that external signals activate partly different neural processes, and displays different anatomical connectivity profiles, than do internal self-reflective processes. While task processes take place in the lateral part of the cortex, internal processes are mainly processed on the medial surface. When performing a cognitive task, such as a rule-switching task where action relevant cues are visually presented, the ventrolateral prefrontal cortex is active primarily in the learning stage, whereas activity in neurons of the premotor cortices and caudate control the task once learned [3,4]. Likewise, there is now substantial support for a role for the anterior medial prefrontal cortex (aMPFC) in processing abstract representations of abilities [5]. When participants reflect upon whether a trait applies to themselves or not, aMPFC activity increases [6,7], or when trait words are processed as compared to non-trait words [8].

Less is known about aMPFC activation during cognitive task performance. Having positive associations to one’s traits is regarded beneficial for performance with the argument that a positive self-view helps with persistence on a task when facing difficulties [9]. A proposed mechanism is that believing in one’s abilities generates a self-fulfilling prophecy, which influences how we strive to achieve our goals [10]. Similarly, associations to being intelligent have been found to increase the confidence in participants’ memory process, as opposed to associations to stupidity [11]. Furthermore, it has been found that when participants believe that their trait is being evaluated, such as their intelligence, error-activation in the paracingulum of aMPFC increases when performing a working memory task [12,13]. This suggests that aMPFC plays a role in performance monitoring from a self-image perspective.

However, these latter studies are inconclusive as to whether it is beneficial for performance accuracy to make associations with cleverness during task performance. In fact, negative effects on performance and motivation as a consequence of a positive self-view have been reported [14,15]. For example, a heightened self-view in the form of high achievement based self-esteem can be a factor behind burnout symptoms [14]. Thus, it may be that the character of the positive self-association is important for performance outcome. There are two principal mind-sets regarding intelligence which have been proposed to be particularly relevant for task motivation and performance; the entity mind-set and the incremental mind-set [16]. A person holding an entity mind-set perceives intelligence as something that is an unchangeable part of their character. An individual with an incremental mind-set on the other hand, regards intelligence as something that can vary with effort and training. In turn, an incremental mind-set gives rise to greater task motivation [17], and greater performance improvements when retested [18]. Studies on feedback have observed that praising intelligence can lead to reduced motivation, and task persistence [19,20]. Although there are several behavioural studies on the topic of praising trait and action, few studies have been looking at underlying neural mechanisms. In an EEG study it was found that having an entity mind-set was related to enhanced P3 response to performance-relevant feedback on errors as compared to incremental theorists. The error-related P3-peak was most likely stemming from aMPFC [18].

Here we have extended the research to involve conflicts more generally by looking at correct trials using a rule-switching task [21]. This task includes two levels of conflict; bivalent rules and univalent rules. In bivalent rule trials, a response symbol has different meanings dependent on the current rule active. It has been found that the response on bivalent correct trials, but not on univalent trials, were influenced by which ability priming (clever or stupid) the participants had received prior to the task [11]. We therefore chose this particular task to study the effect of positive feedback related to trait and feedback related to choice of action. In this functional magnetic resonance (fMRI) study, we investigated if trait focus and task focus could be manipulated with feedback on correct responses in the rule-switching task. The aim was to study the impact of the feedback on perceived experience, performance accuracy, and neural processes with particular focus on the aMPFC. We used task feedback ‘your choice was correct’ that praised participants’ choice, and trait feedback ‘you are clever’ that praised participants’ intelligence in a within-subject design. We hypothesized that the trait feedback would evoke more focus on one’s character with increased neural activation in paracingulate cortex of aMPFC as a result [12,13], and lead to lowered motivation and performance [17,18]. Previous studies show that individuals prone to anxiety tend to fear social feedback [22]. Other studies show that feedback is perceived differently in participants with incremental or entity views on intelligence [18, 23]. We therefore also tested if self-esteem, mood, and growth mind-set influenced how the feedback was processed.

## Materials and Methods

### Participants

Twenty healthy participants (age 24±5.6, 8 females) were scanned with fMRI twice (18.5±6.45 days apart), in a crossover design. Two other participants were tested but excluded, one because of performance below chance-level, the other for attending only one session.

### Ethical statement

Participants gave written informed consent prior to testing. The study was approved by the local ethics committee in Stockholm (EPN), Sweden (Dnr 2014/10-31/2). Participants were neurologically healthy, right-handed, and with Swedish (n = 13) or English (n = 7) as their mother tongue. They performed the task in their native language.

### Rule-switching task

**Fig 1:**
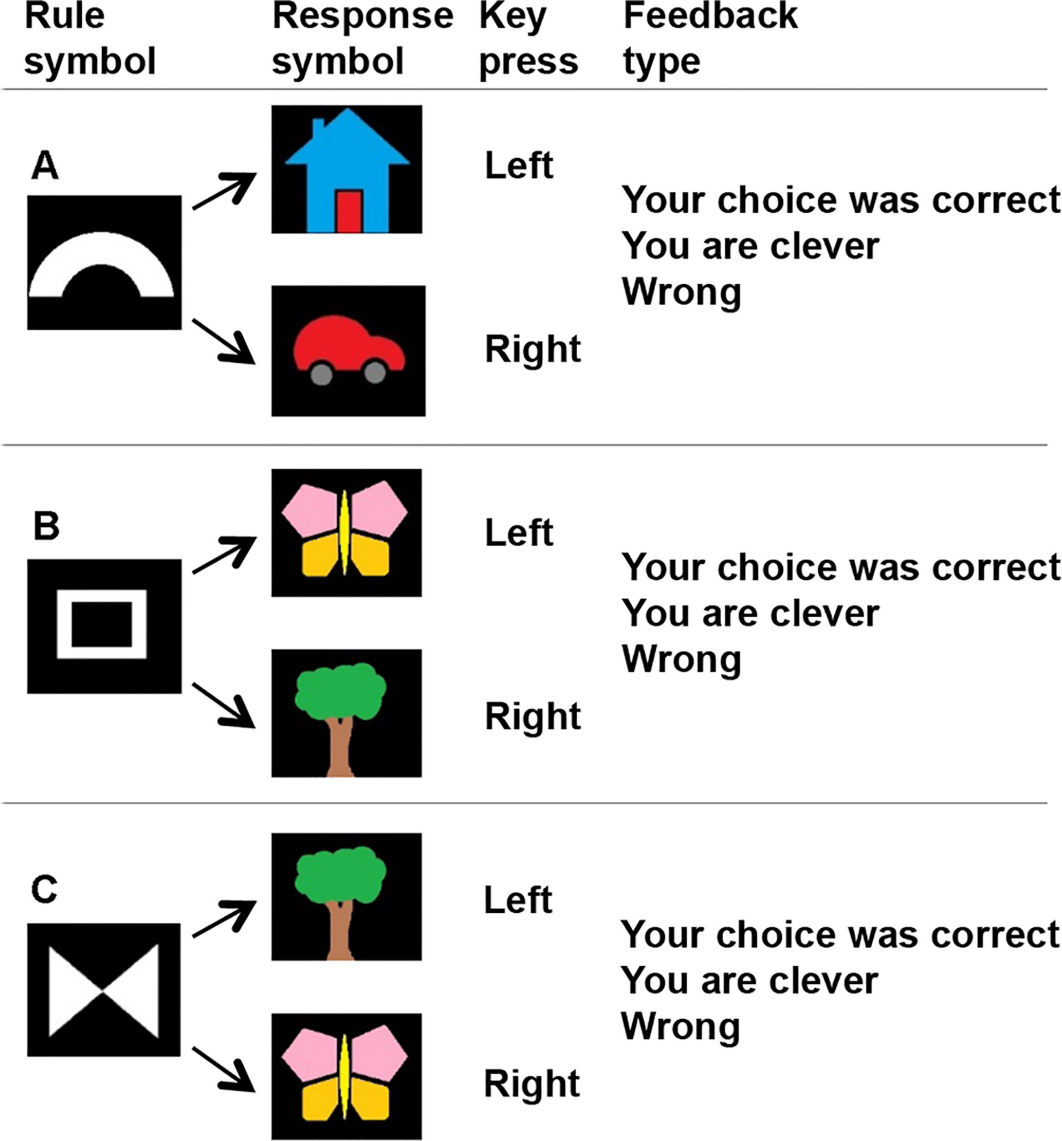
Rule-switching task. A) A univalent rule trial. A rule symbol is presented followed by one of two unique response symbols. B-C) Two bivalent rule trials. Two different rule symbols are used to determine the action to a subsequent response symbol. For example, when seeing the butterfly, either the left or the right key should be pressed depending on preceding rule symbol. A-C) The feedback presented was either task feedback ‘your choice was correct’ or trait feedback ‘you are clever’ for correct responses, and 'wrong' for incorrect responses.

The participants took part in four scanning sessions, two sessions per visit, in a computer based rule-switching task [21]. In the task, a rule symbol was presented on a computer screen for 1000ms, followed by a blank screen for 500ms before a response symbol appeared. During the presentation of the response symbol, the participants had a window of 2500ms to respond, and received feedback immediately after the motor response (Fig 1). This was followed by jittered delay of 1.5, 4 or 6 sec before presentation of the next rule symbol. This jittered delay was introduced so as to separate (de-correlate) neural activations between the different trials, i.e. neural activations evoked by the feedback in one trial and the neural activations evoked by seeing the rule symbol in the consecutive trial. The participants responded to symbols that could be either univalent or bivalent by pressing the left or right button on a button box. The univalent trials were associated with fixed responses, e.g. when a symbol of a bow (rule symbol) is followed by a house (response symbol) a left key press is the correct answer, whereas a right key-press is correct when a bow is followed by a car (Fig 1a). Bivalent trials refer to visual pictures that were associated with different responses depending on one of the two rules possible. For example, if a rule symbol consisting of a square is followed by the response symbol of a butterfly, the participants should press the left button. On the other hand, if the rule symbol is a triangle and is followed by the same response symbol, a butterfly, the participant should press the right button (Fig 1b and 1c). The task consisted of a distribution of 70% bivalent trials and 30% univalent trials with a distribution combination in accordance with earlier studies [11,21]. This distribution yields a fairly equal frequency of each rule; 35% presentations of each for the two bivalent rules (Fig 1b and 1c), and a 30% occurrence of the univalent rule (Fig 1a). Univalent trials served as controls and were later in the fMRI analysis subtracted from bivalent trials in order to study brain activation related to conflicting trials. The univalent trials balance the comparisons for visual input, and motor output. In addition, by subtracting brain activation related to univalent rule trials from brain activation related to bivalent rule trials, for each participant at each visit, we reduce the influence of confounding effects, such as equipment and participant related effects, which may vary between the two scanning visits. The distribution of univalent and bivalent trials was identical between the two scanning visits. The participants were presented with a set of different symbols on each visit.

#### Practice

The task, with feedback, was practiced for 12 trials on a PC computer in a testing room prior to fMRI scanning. If the participant’s accuracy was <60% they received task instructions again and practiced until achieving ≥60% correct. Two participants gave <60% correct responses on their first practice trial. Inside the scanner, the participants were familiarized with the button box and the visual display for another 12 trials before data collection started.

#### Feedback

Each scanning visit comprised of a feedback session (FS) followed by a no feedback session (NFS). During the FS, the feedback was presented immediately after each key-press. The feedback was either task feedback ‘Your choice was correct’/’Du valde rätt’ (Swedish), or trait feedback ‘You are clever’/‘Du är smart’ (Swedish) for corrects and ‘Wrong’/‘Fel’ (Swedish) for incorrect responses. During the NFS there was no feedback given neither on corrects nor on errors. The FS consisted of 100 trials and the NFS consisted of 50 trials. Only one of the feedbacks was given per visit. The order of which feedback type (task/trait) was received on their first and second visit was counterbalanced among participants. The consequence of the counterbalance is that the time confound of NFS always following FS does not affect the comparison between the two feedback conditions ‘You are clever’ and ‘Your choice was correct’. Moreover, the identical number of trials between the two feedback conditions, and the fact that we analyze the data with a 2x2 ANOVA, gives the unequal number of presentations between FS and NFS, and univalent and bivalent trials, negligible impact on the results [24].

### Questionnaires

A minimum of one day before the experiment (mean 6.5±9.2 days), all participants filled in the following questionnaires using Google forms; Burns depression inventory [25] and Rosenberg self-esteem score [26], and four selected questions regarding intelligence mind-set orientation [17]. The assessment of mind-set was calculated by subtracting the scores of the two entity questions from the scores of two incremental questions. The questions were answered by numbers 1–6; 1-Strongly disagree, 6-strongly agree. After subtraction, negative scores indicated agreement with an incremental mind-set and positive scores indicated agreement with entity mind-set.

### Motivation ratings

At three times during scanning, the participants rated their motivation, stress, and task difficulty by answering three questions asked by the experimenter; How motivated are you to continue with the task?; How stressed do you feel right now?; How difficult did you find the task? Ratings were of 1–10; 1-very calm/unmotivated/easy, 10-very stressed/motivated/difficult. The questions were asked before the cognitive task, after FS, and after NFS (difficulty ratings were not given before the task). These are presented in Table 1. After the experiment, the participants wrote down how they experienced the feedback during the different sessions with the questions; What did you think about the test with feedback? What did you think about the test without feedback? They were subsequently debriefed about the purpose of the experiment.

**Table 1:** Behavioural data.

Measure	Feedback	Session	Score	SD
Accuracy (percent correct)	Trait	FS	91.6	8.0
	-	NFS	95.4	8.2
	Task	FS	90.6	7.8
	-	NFS	97	2.9
Accuracy (bi)	Trait	FS	88.4	10.9
	-	NFS	94.4	9.2
	Task	FS	87.2	10.2
	-	NFS	96.5	3.0
Accuracy (uni)	Trait	FS	97.8	4.8
	-	NFS	97.1	7.0
	Task	FS	97.2	5.6
	-	NFS	97.9	3.5
RT correct (ms)	Trait	FS	686.7	169.8
		NFS	629.3	167.8
	Task	FS	692	146
	-	NFS	637.2	152.8
RT correct (bi)	Trait	FS	744.2	208.2
		NFS	624.9	165.2
	Task	FS	738.4	172.2
	-	NFS	638.9	158.6
RT correct (uni)	Trait	FS	590.5	129.7
		NFS	639.0	176.4
	Task	FS	620.6	128.3
	-	NFS	633.8	148.4
Motivation scores (1–10)	Trait	before FS *	7.3	2.7
	Trait	after FS	6.6	2.5
	-	after NFS	5.9	2.9
	Task	before FS *	7.6	2.6
	Task	after FS	7.4	2
	-	after NFS	6.3	2.6
Stress scores (1–10)	Trait	before FS *	2.5	1.7
	Trait	after FS	2.5	1.3
	-	after NFS	2.2	1.6
	Task	before FS *	2.2	1.2
	Task	after FS	2.2	1
	-	after NFS	1.8	0.78
Difficulty scores (1–10)	Trait	after FS	2.5	1.4
	-	after NFS	2.4	1.5
	Task	after FS	2.8	1.5
	-	after NFS	2.2	1

Participants’ accuracy, reaction time on correct responses (RT) and subjective ratings of motivation, stress and difficulty. Average scores are presented for the feedback sessions (FS) and the no feedback sessions (NFS) for task and trait feedback respectively. Motivation and stress scores were assessed at three time points for each feedback type. Difficulty scores were assessed after FS and NFS. The scales ranged from 1–10 where low number indicated low motivation/stress/difficulty.

* = practice was made with corresponding feedback.

### Behavioural Data Analysis

The data was presented using Cogent software (UCL, London, UK) which is running in Matlab (r2010a, The Math Works, MA, USA). Accuracy (% correct), reaction time (RT), motivation scores, stress scores, and difficulty scores were analysed with repeated measures ANOVAs, and Student’s paired t-tests for post-hoc comparisons in SPSS (SPSS Inc, Chicago, USA). We report behavioural results from a 2x2 repeated measure ANOVA with the factors feedback (task/trait) and time (FS/NFS). All the behavioural analyses were controlled for the order of which the participants received each feedback type (day of testing). We further investigated if there were any correlations between the outcome measures; accuracy, RT for correct responses, motivation scores and the Burns/Rosenberg questionnaires, using Pearson correlations in SPSS.

### Imaging acquisition

Functional imaging data were acquired using a 3T GE scanner (Discovery MR750, GE). Functional images sensitive to blood oxygen-level dependent (BOLD) contrasts were acquired using echo-planar imaging as T2*-weighted images. Whole brain image volumes were built up from contiguous oblique slices (n = 40), flip angle 90°, TR = 2600ms; TE = 30ms; FOV 28,8cm; matrix size 64*64; voxel size 2x2x2mm, slice thickness 3.0mm with 0.5mm gap. A high-resolution 3D gradient-echo, T1-weighted anatomical image was also collected for each participant. We used an 8-channel head coil.

### Brain imaging data analysis

The fMRI data was analysed in SPM12b (www.fil.ion.ucl.ac.uk/spm/, London, UK) [27]. The first six volumes were discarded and the remaining volumes realigned to the first volume to correct for head movements. Subsequently, the volumes were co-registered and normalized to standard space using the Montreal Neurological Institute reference brain. The time series were smoothed spatially with an isotropic Gaussian filter of 8 mm full width at half-maximum.

The fMRI data was modeled as an event-related design with regressors corresponding to bivalent, univalent, correct, and incorrect trials for the conditions with immediate feedback (trait/task) (FS) and for the subsequent conditions without feedback (NFS), for each scanning visit. The events of the trials (bivalent/univalent and correct/incorrect) were time-locked to the presentation of the rule symbol, and the key response.

Whole brain statistical parametric maps were calculated for these event-related condition-specific effects with the general linear model. The vectors were convolved with a canonical hemodynamic response function, and the data were high-pass filtered with a frequency cut-off of 128 sec.

The subject-specific effects were taken up to a second-level full-factorial analyses based on summary statistics from the contrast-images created on the first level, using 2 factors with 2 levels each; rule (univalent/bivalent) and condition (task/trait). Due to the low number of error trials we excluded the factor outcome.

We investigated the main effect of bivalent trials, main effect of condition (task/trait), and interactions between rule (bivalent/univalent) and condition (task/trait) where we present both cross-over (co) and one-way (ow) results. This was done for both the FS and the NFS. We also investigated the interaction between condition (task/trait) and time (FS/NFS). The analyses were focused on the event that was locked to the time of seeing the rule symbol, as well as at the time of pressing the key i.e. seeing the feedback.

We also investigated if BOLD was related to motivation scores, stress scores, difficulty scores as measures in between the FS and NFS, and performance accuracy for the following conditions: BOLD at the event of seeing the bivalent rule symbol, and at the event of seeing the feedback, in the trait feedback and task feedback conditions during the FS and NFS. For this purpose, we added each rating separately as a covariate in second level SPM analyses. Because we tested four different outcome measures, we then corrected the p-values for multiple comparisons with Bonferroni’s corrections.

In order to test for significance, we carried out whole brain Family Wise Error corrected statistics (p<0.05) (Table 2). In Table 3, we also present results on p<0.001 uncorrected level. To investigate if the activations found on the uncorrected level fell within task specific areas, we applied a mask generated from the activation pattern of the contrast: Main effect Rule (bivalent vs univalent) whole brain corrected. To test the hypothesis of feedback sensitive activity in aMPFC we carried out restricted region of interest (ROI) analyses based on previous findings of paracingulate cortex (-10 50 30) [12,13]. We report results corresponding to ROIs in the shape of a sphere with a radius 6mm, p-value <0.05 corrected for multiple comparisons (FWE). Anatomical locations were verified using the atlas of Duvernoy [28].

**Table 2:** Brain activations at a statistical threshold of p<0.05 FWE-whole brain corrected.

Rule symbol (FS)	Contrast	Area	Side	x	y	z	t-score
trait + task	bi-uni	Mid frontal g	L	-26	8	50	5.13
		Mid ACC	R	10	22	36	5.71
		Insula	R	34	22	2	6.57
		Caudate	L	-14	6	0	7.62
		Caudate	R	12	8	-4	7.79
		Putamen	R	26	18	-2	6.22
		Thalamus	R	6	-14	8	5.45
		Precuneus	L	-8	64	42	5.44
		Angular g	R	-38	-54	36	5.26
		Calcarine s	L	-12	-74	14	5.22
Trait > task	co bi-uni	Middle front g	R	26	6	36	5.09
		Caudate	R	24	20	18	5.39
Trait > task	ow bi-uni	Caudate/putamen	L	-22	18	-12	5.09
Task>trait		n/a					

Brain activations at whole brain FWE corrected (p<0.05) level. Activations were seen when the participants were viewing the bivalent rule symbols in the conditions where they got the feedback ‘You are clever’. Bi—bivalent trials, uni—univalent trials, co—crossover interaction, ow—oneway analysis.

**Table 3:** Brain activations at a statistical threshold of p<0.001 uncorrected. Brain activation at uncorrected (p<0.001) statistics for feedback session (FS) and no feedback session (NFS) at the event of seeing the rule symbols and at the event of making a key press/seeing the feedback ‘you are clever’ and ‘your choice was correct’, for bivalent trials (bi) and univalent trials (uni) trials. co—cross-over analysis, ow—one way analysis.

**Rule symbol (FS)**	**Contrast**	**Area**	**Side**	**x**	**y**	**z**	**t-score**
trait > task	co bi—uni	Middle front g	R	26	6	36	5.09
		Central s	R	20	-44	50	4.81
		Caudate	L	-16	14	22	3.32
		Caudate	R	24	20	18	5.36
		Cerebellum	L	12	-66	-40	3.6
trait > task	ow bi—uni	Mid front g	L	-28	6	48	3.78
		Mid front g	R	26	2	40	3.95
		ACC	L/R	10	24	36	4.66
		ACC	L/R	-12	20	28	4.07
		Orb front g	R	16	54	-6	3.9
		Caudate/putamen	L	-22	18	-12	5.09
		Caudate/putamen	R	14	10	0	4.91
		Precuneus		-8	-64	42	4.7
		Inf parietal g	L	-36	-56	32	3.51
		Mid occipital g	R	22	-76	12	3.99
		Vermis		-12	-48	-40	3.5
trait > task	bi + uni	Insula	R	38	8	-8	3.5
		Insula	R	32	-12	20	3.31
		Amygdala	L	-20	-4	-16	3.67
task > trait	co bi—uni	Insula	L	-46	4	-10	3.29
task > trait	ow bi—uni	Central g	L	-22	-42	74	3.68
		Globus Pallidus	L	-14	2	-4	3.83
		Globus Pallidus	R	10	6	-6	3.84
**Rule symbol (NFS)**	**Contrast**	**Area**	**Side**	**x**	**y**	**z**	**t-score**
trait > task	co bi—uni	Frontopolar g	M	-2	64	-8	3.38
		Caudate	L	-10	10	20	3.77
		Angular g	L	-48	-68	26	3.48
		Angular g	R	48	-60	18	3.47
		Inf temporal g	L	-62	-10	-16	3.19
		Cerebellum	R	40	-74	-38	3.52
trait > task	ow bi-uni	Mid front g	L	-24	24	58	3.81
		Precentral g	R	18	-18	56	4.9
		Sup marg g	L	50	-60	16	3.94
trait > task	bi + uni	Inf frontal g	L	-58	6	36	3.12
		Precentral g	R	26	-14	56	3.24
		Precuneus	R	12	-58	50	3.51
task > trait	co bi—uni	Ant calcar s	L	-16	-46	8	3.57
**Response symbol (FS)**	**Contrast**	**Area**	**Side**	**x**	**y**	**z**	**t-score**
trait > task	co bi—uni	Inf frontal g	R	48	32	-2	3.32
		Sup temp g	L	-46	6	-10	3.24
trait > task	ow bi—uni	Inf frontpol s	R	28	56	-4	3.43
		Inf front g	R	50	15	-8	3.4
		Lat cerebellum	R	42	-68	-30	3.39
trait > task	bi + uni	Central s	R	24	-46	54	3.59
		Temporal pole	R	46	16	-36	3.53
		Mid occip g	L	-34	-80	-6	3.16
task > trait	co bi—uni	Cuneus	L	-8	-98	14	3.25
task > trait	ow bi—uni	Putamen	R	26	-10	20	3.77
		Lat cerebellum	L	-28	-64	-40	3.74
		Lat cerebellum	R	30	-62	-42	3.76
**Response symb (NFS)**	**Contrast**	**Area**	**Side**	**x**	**y**	**z**	**t-score**
trait > task	co bi—uni	Ant calcar s	L	-16	-46	8	4.47
		Cerebellum	L	-24	-48	-38	3.32
trait > task	ow bi—uni	Occ g	L	-28	-80	0	3.79
		Occ g	R	22	-82	2	3.59
		Vermis	R	12	-50	-22	3.43
trait > task	bi + uni	Sup frontal g	R	-16	22	42	3.92
		Sup/med front g	R	-8	32	48	2.42
		Angular g	R	52	-36	8	3.24
task > trait	co bi—uni	Caudate	L	-10	12	18	4.01
		Insula	L	-40	18	12	3.69
**Rule symb (FS-NFS)**	**Contrast**	**Area**	**Side**	**x**	**y**	**z**	**t-score**
trait > task	co bi	Thalamus	R	26	-10	22	3.74
trait > task	ow bi	Mid frontal g	R	20	14	68	4.47
		Angular g	L	-42	-48	36	3.76
		Thalamus	R	26	-12	22	3.51
task > trait		n/a					
**Resp symb (FS-NFS)**	**Contrast**	**Area**	**Side**	**x**	**y**	**z**	**t-score**
trait > task	ow bi	Rost ACC		-4	40	20	3.94
		Precentral g	L	-56	-18	44	3.82
task > trait	co bi	Central s	L	-60	-6	40	3.57
		Putamen	R	32	10	0	3.52
		Vermis		-4	-72	-24	3.98
		Cerebellum	R	24	-64	-26	3.74
task > trait	ow bi	Mid front g	R	48	8	50	3.54
		Vermis		-10	-36	-26	3.94

## Results

### Behavioural results

#### Questionnaires

We found a correlation between Burns depression scores and Rosenberg self-esteem scores (Pearson correlations, r = 0.87, p = 0.001) where the lower the self-esteem the more negative mood. When correlating ratings on Burns depression inventory and Rosenberg’s self-esteem scale with motivation ratings during the scanning, performance accuracy, or RT, we did not find any significant correlations (Pearson correlations r<0.1, p>0.05). When analyzing participants’ incremental and entity views on intelligence we found twelve participants holding an incremental view and seven participants holding an entity view. One participant got a score of zero, meaning neither nor. There was no significant interaction between mind-set and feedback on accuracy (F_(1,16)_ = 0.387, p = 0.543, two-tailed), nor RT, (F_(1,16)_ = 0.861 p = 0.769, two-tailed). There was a significant interaction between mind-set and feedback when looking at motivation (F_(1,16)_ = 6.040, p = 0.026, two-tailed). Motivation score in participants with an entity mind-set was 5.43±3.04 after trait-feedback (FS) and 8.14±1.86 after action feedback (FS) (t_(18)_ = 2.03, p = 0.09, two-tailed). Motivation score in participants with an incremental mind-set was 7.25±2.05 after trait feedback (FS) and 6.91±2.15 after action feedback (FS), (t_(18)_ = 0.87, p = 0.4, two-tailed).

#### Accuracy

**Fig 2:**
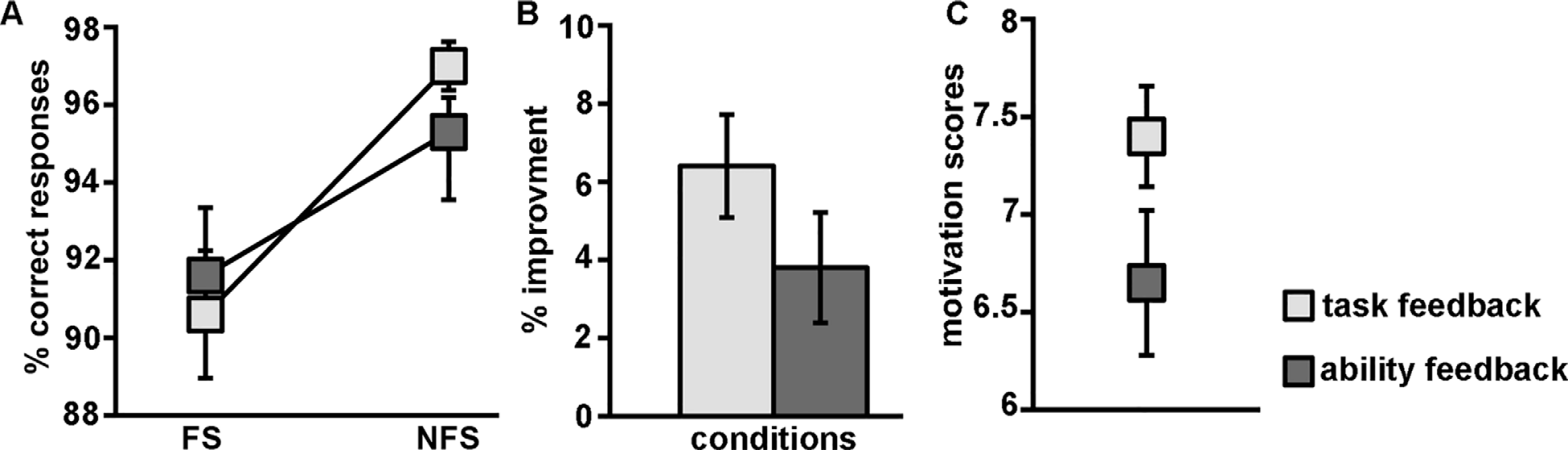
Performance improvement and motivation scores for task and trait-feedback during FS and NFS. A) Both feedback conditions resulted in performance improvement from FS to NFS. B) Task-feedback led to greater improvement compared to trait-feedback (NFS vs. FS). C) The participants were more motivated to continue with the task after task-feedback compare to trait-feedback.

The behavioural data revealed a significant effect of time (FS or NFS) on accuracy (F_(1,18)_ = 6,24, p = 0.02, Fig 2a and 2b). The post-hoc analysis showed that accuracy was higher in the NFS than the FS both in the trait-feedback sessions (t_(19)_ = 4.7, p = 0.017) and in the task-feedback sessions (t_(19)_ = 2.6, p = 0.0001). Overall, accuracy was significantly higher for univalent trials compared to bivalent trials trials during FS (F_(2,18)_ = 31.18, p = 0.001) and during NFS (F_(2,18)_ = 8.796, p = 0.008) (Table 1). We observed an effect of feedback when looking at accuracy improvement (NFS accuracy—FS accuracy) from FS to NFS, over all correct trials (Fig 2a and 2b). Task-feedback lead to a greater improvement than trait-feedback when looking at time(FS/NFS)*feedback (task/trait), (F_(2,18)_ = 3.550, p = 0.038, one-sided), where task-feedback had an average increase of 6.4% SD±6.5, and trait-feedback gave an increase of 3.8% SD±6.0. Looking only at the bivalent trials, again there was a significant improvement in favour of task feedback (time(FS/NFS)*feedback (task/trait), F_(2,18)_ = 3.907, p = 0.032, one-sided). This effect was absent for univalent trials (time(FS/NFS)*feedback (task/trait), F_(2,18)_ = 0.423, p = 0.26, one-sided) (Table 1).

#### Reaction time

There was a significant effect of time (FS/NFS) on RT for correct responses (F_(2,18)_ = 29.32, p = 0.0001). RT was slower in FS than NFS for both task and trait-feedback. Bivalent trials were slower than univalent trials in FS (F(2,18) = 51.1, p = 0.001), but there was no difference between bivalent and univalent trials in NFS (F(2,18) = 0.34, p = 0.57). There was a trend for a difference between condition (task/trait) and trial type (bivalent/univalent) (F(1,18) = 3.305, p = 0.086, two-sided), where the participants tended to slow the bivalent trials in the trait feedback condition (Table 1).

#### Motivation ratings

There was a main effect of feedback on motivational scores (F_(1,18)_ = 6.244, p = 0.022). After the task-feedback FS, motivation scores were significantly higher than after trait-feedback FS (task-feedback: 7.4±2.03; trait-feedback 6.65±2.50, F_(1,18)_ = 6.2, p = 0.01,one-tailed) (Fig 2c). There was no main effect of time (FS/NFS) on motivational scores (F_(1,18)_ = 0.71, p = 0.50), nor a difference in motivation before the scanning started between the two visits (t(_19_) = 1.65, p = 0.21) (Table 1).

#### Stress ratings

There was a significant effect of feedback on stress scores as measured after the FS and NFS (F_(1,18)_ = 14.69, p = 0.001), where task-feedback induced less stress than trait-feedback. There was also a significant interaction between feedback and order (F_(1,18)_ = 12.13, p = 0.003), where stress scores were higher on the first visit both after FS and NFS compared to the second visit, when receiving trait-feedback. There was no effect of time (FS/NFS) on stress scores (F_(1,18)_ = 2.355, p = 0.13).

#### Difficulty ratings

There was no effect of feedback on difficulty scores, (F_(1,18)_ = 0.22, p = 0.65), nor time (FS/NFS) (F_(1,18)_ = 1.32, p = 0.27).

#### Debriefing

The following data was revealed from the debriefing about feedback experiences. In regard to task-feedback (FS), 75% of the participants were positive, 20% negative, and 5% neutral. In regard to NFS following the task-feedback, 70% were positive, 25% negative, and 5% neutral. In regard to trait-feedback (FS), 30% were positive, 45% negative, and 25% neutral. In regard to NFS following trait-feedback, 60% were positive, 20% negative, and 20% neutral. Thus, trait related feedback was most disliked. Positive views typically included ‘nice’, ‘motivating’, or ‘it was good’. Negative views typically included ‘distracting’, ‘annoying’, or ‘repetitive’.

#### Brain activation

**Fig 3:**
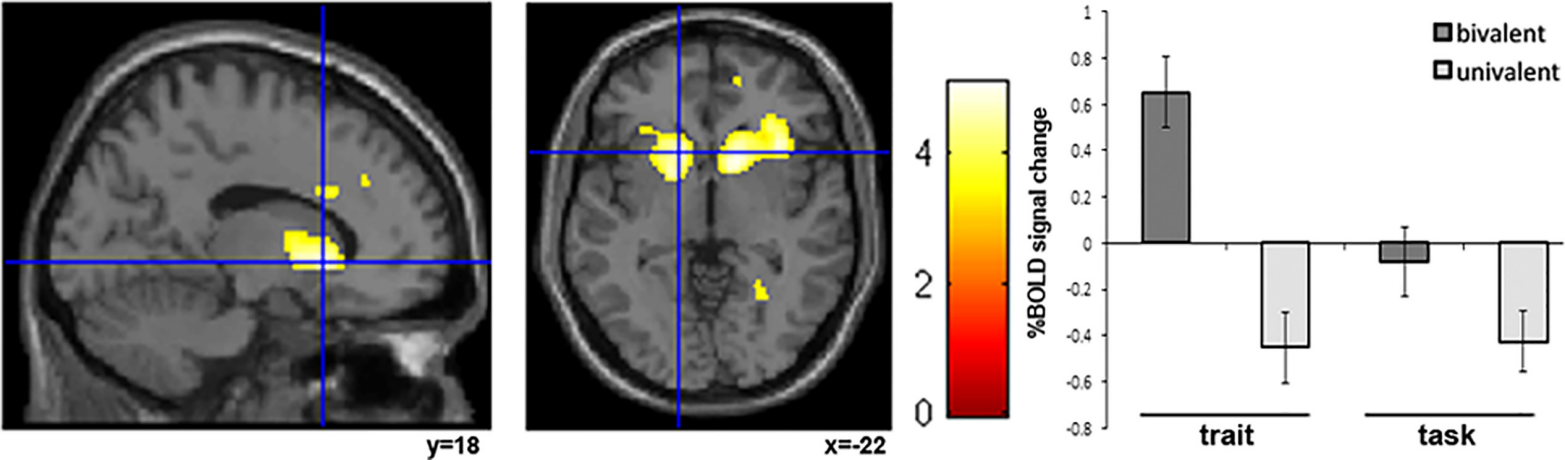
Brain activation in left anterior caudate nucleus when seeing rule symbols. A) Activity increased at the event of seeing the bivalent rule symbols in FS for ‘you are clever’. B) % BOLD signal change for each condition and rule type.

The left anterior caudate nucleus was more active at the event of seeing the bivalent rule symbol in the FS in the condition where the participants were presented with the feedback ‘you are clever’, as compared to the condition where they received the feedback ‘your choice was correct’. This was revealed both in a cross-over and in a one-way analysis between rule (bivalent vs. univalent) and condition (trait vs. task) (24 20 18, t = 5.39, p = 0.014; -22 18 -12, t = 5.09, p = 0.036 FWE whole brain corrected) (Fig 3, Table 2). Brain activations at a lower statistical threshold (p<0.001 uncorrected) are presented in Table 3. We note that caudate activation was not seen to differ between the two feedback types when contrasting FS bivalent trials to NFS bivalent trials (trait [FS-NFS] vs. task [FS-NFS] and vice versa) (Table 3). This suggests that the effect of feedback is more pronounced on bivalent trials and less so on univalent trials. This finding is in line with the accuracy and RT data, which show feedback dependent differences for bivalent trials but not univalent trials.

None of the ratings regarding perceived quality (stress/motivation/difficulty) correlated with BOLD in this area for the feedback condition ‘you are clever’.

**Fig 4:**
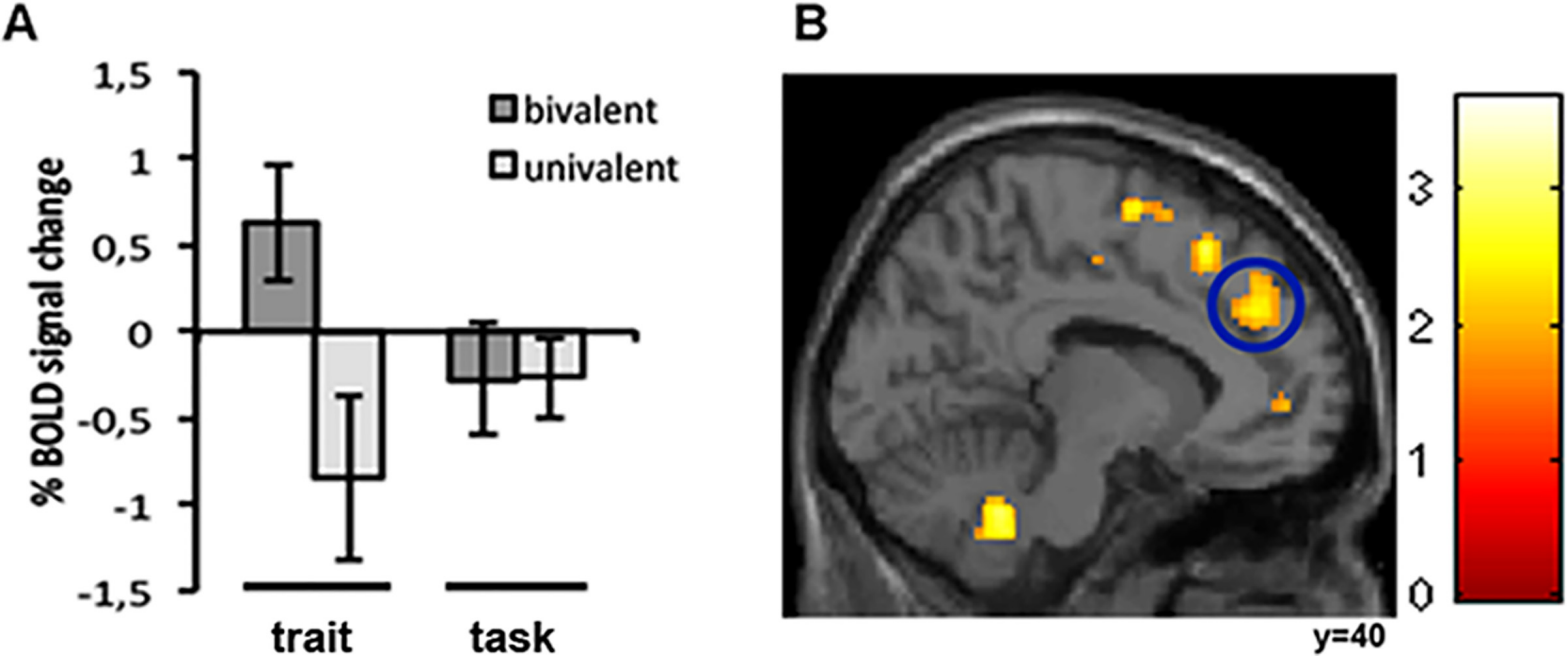
Brain activation in anterior paracingulate cortex when seeing rule symbols. A) % BOLD signal change for each condition and rule type. B) Activity increased in the trait condition compared to task condition.

When investigating activity of the paracingulate cortex of aMPFC specifically, again we found increased activation in particular when the rule symbol was presented for the feedback condition ‘you are clever’. Seeing the rule symbol during the FS was reflected as enhanced activity when investigating main effect trait vs. task (bivalent + univalent) (12 52 28, t = 2.46, p<0.05 corr 4mm ROI) (Fig 4a and 4b). Seeing the rule symbol during the NFS was reflected as enhanced activity when investigating the interaction between rule (bivalent vs. univalent) and condition (trait vs. task) (6 50 26, t = 2.76, p<0.05 corr ROI), as well as in the simple contrast trait bivalent rule vs. trait univalent rule (6 50 28, t = 3.08, p<0.02 corr ROI). None of the quality ratings correlated with BOLD in this area for the condition ‘you are clever’. Instead, there was a significant positive correlation between BOLD and performance accuracy at the event of seeing the bivalent rule symbol, in the NFS following ‘your choice was correct’ (16 50 30, t = 4.26, p<0.008 Bonferroni corrected, ROI).

At the time of seeing the feedback, there was no activation surviving the conservative statistical threshold of correcting for multiple comparisons in the whole brain. We observe however, that at the threshold p<0.001 uncorrected, there is more activity when seeing the feedback ‘you are clever’ as compared to ‘your choice was correct’ (Table 3). Increased activations when seeing the feedback ‘you are clever’ vs. ‘your choice was correct’ in bivalent trials as compared to univalent trials, were found in right inferior frontal gyrus (48 32 -2, t = 3.32) and left superior temporal gyrus (-46 6 -10, t = 3.24). Increased activation when seeing the feedback ‘your choice was correct’ vs. ‘you are clever’ in bivalent trials as compared to univalent trials, was observed in cuneus (-8 -98 14, t = 3.25). In the NFS, at the time of the response, which is the time when the feedback was presented in the first part of the scanning session, activation increased in the visual cortex (anterior calcarine sulcus, -16 -46 8, t = 4.47) and cerebellum (-24 -48 -38, t = 3.32). This was seen for the contrast comparing activation during ‘you are clever’ to ‘your choice was correct’ in bivalent trials as compared to univalent trials. On the other hand, the condition ‘your choice was correct’ vs. ‘you are clever’ (bivalent trials vs. univalent trials) gave rise to left caudate (-10 12 18, t = 4.01) and left insular cortex (-40 18 12, t = 3.7) activation increase.

## Discussion

We found that trial-based written feedback influenced participants’ perceived experience and their performance. The participants were more motivated to continue with the task after the FS ‘your choice was correct’ than after the FS ‘you are clever’. They also perceived less stress in the ‘your choice was correct’ condition, and showed a significantly greater improvement in accuracy from FS to NFS. In the debriefing after the testing, nine of the participants expressed negative views about the feedback ‘you are clever’, whereas only four participants reported negative views about the task feedback. Thus, although both feedback types informed the participants about their correct outcome, the one addressing a trait gave rise to a more negative experience. This result is in line with a number of previous behavioural studies observing that trait-focused feedback has a negative effect on mainly motivation, but also on performance [16–18,29,30].

Interestingly, in the ‘you are clever’ condition we observed enhanced activation for the bivalent rule trials in particular. Mostly at the time when the bivalent rule symbol was presented. This means that the feedback-induced effects influence task monitoring. When looking at the simple, univalent trials the difference between the feedback types cease to appear. For example, we observed significantly greater activation in caudate nucleus for bivalent trials in the condition ‘you are clever’ than in the condition ‘your choice was correct’, but there was no difference between the two conditions when it came to the univalent trials. Related finding was made when looking at the behavioural data; RT and accuracy, where no difference was seen between feedback conditions in how univalent trials were treated, but more so when studying bivalent trials. We also looked at the bivalent trials during FS when using NFS bivalent trials as a control, to investigate if caudate was more active as a function of the actual feedback. There was no support for such a process, which strengthen the notion that more complex bivalent, rather than simple univalent trials, are targets for effects induced by the feedback. Similar observations have been made previously in that trait associations are particularly influential on neural activity and behaviour during events of uncertainty, such as errors or bivalent rule cues [11–13,18]. The results suggest that the ‘You are clever’ feedback does not induce active and constant thoughts regarding one’s trait. Rather, it seems that the feedback influences processes that impacts on neurotransmitter levels so that, in moments of uncertainties, the neurons are signaling differently. The most likely neurons to be affected by the feedback are the one’s sensitive to dopamine. This is so because the caudate nucleus is known to innervate high densities of dopamine sensitive neurons [31,32] and uncertainty is reflected in dopamine signals [33, 34].

Uncertainty is a measure of the quality of the participant’s own estimate of the data [35]. Poor quality can be due to that not enough data has been collected [35], or because of poor confidence in the memory for data presented [11]. The latter would be the likely explanation in the present context as the two feedback conditions ‘You are clever’ and ‘Your choice was correct’ did not differ in the number of data presentations. Reduced confidence in memory could be due to factors such as anxiety, more demanding interpretation of the feedback, or deviation from task focus. Increased caudate activation has previously been observed when individuals anticipate the value of outcomes [36], where participants with social anxiety show higher activation in caudate nucleus in anticipation to a performance outcome compared to controls [37,38]. Individuals may be more sensitive to information regarding their character (clever as opposed to correct action). According to the processing efficiency theory [39], anxiety makes us use compensatory strategies, such as increased effort, in order to perform well. Another possibility to the observed feedback effects could be that the participants have to engage in a two-step interpretation process in the ‘you are clever’ condition. They need to translate ‘you are clever’ into ‘I made the correct choice’, which requires extra neuronal resources. It has previously been observed that trial-to-trial feedback with low additional information content distracts task attention [40]. A third possible explanation to the observed effects of the feedback is that of a model in social psychology which proposes that attentional shift from action to trait leads to performance impairments due to reduced cognitive resources for the task [41]. For example, working memory capacity has been found to be influenced by how we think about our abilities [42]. Here, the authors induced the stereotypic belief that women are inferior to men in mathematics. Participants receiving the stereotypic framing in this study did not differ in math scores compared to a control group, but the men and women in the primed group took longer time on the math equations and had higher concerns about how the researcher would rate their performance compared to the control group. The main aim of the study however, was to look at how participants performed on a working memory task which was incorporated between the math equations. Results on the working memory task revealed that the women in the stereotypic group recalled fewer words when compared to a control group of women. This implies that the threat of belonging to a negative stereotype makes participants reflect upon themselves and their abilities, which then interferes with attentional resources needed for the working memory task. Furthermore, Bengtsson and Penny [11] developed a model of the rule-switching task after participants had been primed with association to ‘clever’ and ‘stupid’. The model is based on the current understanding that neuronal populations estimate prior probabilities of outcomes and their expected value. In short, the model stipulates that the individual, by using Bayesian inference, attributes the probability of the cause of a response outcome (correct/error) to either a me-process or a task-process. Associations to ‘clever’ led to enhanced me-focus during correct responses.

When investigating activity in paracingulate cortex, we made two observations. Firstly, we found that this area was more active in the ‘you are clever’ condition during the FS. Secondly, in the absence of feedback, in the NFS following ‘your choice was correct’, we found that participants’ performance accuracy correlated positively with BOLD here. These observations were made at the event when they were viewing the bivalent rule symbols. Our results strengthen the notion that this area is involved in self-focused processes during cognitive performance [12,13]. However, these new findings suggest that activation in paracingulate cortex is sensitive to whether external information about oneself is presented, or if the individual relies on internal self-monitoring.

We further investigated if aspects such as self-esteem, mood, and growth mind-set impacted on how the feedbacks were processed. We found no support for that self-esteem or mood influenced the perception of the feedback. Nor did we observe any effects on accuracy and reaction time in the interactions between feedback and growth mind-set. We noted that there was a tendency for task feedback (your choice was correct) to be particularly beneficial for motivation in individuals with an entity view on intelligence. This could be interpreted in line with the predictions of Dweck et al., (2004) [23] stipulating that entity theorists are more concerned about their outcome whereas incremental theorists are more interested in the knowledge acquired. Although what has been observed previously is a difference between the two mind-sets when processing negative feedback [18]. The current sample sizes are very small and therefore we are making observations but not drawing any conclusions regarding how a growth mind-set is influencing this type of feedback.

We made the decision to scan the participants with 2–3 weeks apart, randomly distributed between the two conditions. This time span was implemented to reduce the influence of the memory from previous visit on the current. It may be that the time factor influences the results because of for example equipment and participant related effects. However, in the brain imaging analysis, for each participant and visit, we subtracted brain activation related to univalent rules from brain activation related to bivalent rules, which lessens the influence of such confounding effects that may vary between the two scanning visits.

### Conclusion

This work sheds light on differences between the self-reflections; trait and action. It turned out that trait feedback was less beneficial for motivation and performance improvement. As a result, activity in caudate nucleus was enhanced in conflicting trials in the trait feedback condition particularly. We observed further that anterior paracingulate cortex activation was sensitive to whether self-reflection was imposed by external feedback or generated from internal processes, and that the latter activity supported performance when followed after task feedback. It should be noted however that these brain areas, which we found differentially active in the two feedback conditions, were most likely not directly differentiating between trait and action per se. Instead, their activation reflected the influence the feedback had on attention or task uncertainty.
